# A Novel Mixed-Methods Platform Study Protocol for Investigating New Surgical Devices, with Embedded Shared Learning: Ibra-net Breast Lesion Localisation Study

**DOI:** 10.29337/ijsp.136

**Published:** 2021-04-16

**Authors:** Hannah L. Bromley, Rajiv Dave, Chris Holcombe, Shelley Potter, Anthony J. Maxwell, Cliona Kirwan, Senthurun Mylvaganam, Suzanne Elgammal, Jenna Morgan, Sue Down, Tahir Masudi, Amtul Sami, Nicola Barnes, James Harvey

**Affiliations:** 1 Nightingale Breast Centre, Manchester University NHS Foundation Trust, United Kingdom; 2 Health Economics Unit, University of Birmingham, United Kingdom; 3 Breast Unit, Royal Liverpool University Hospital, United Kingdom; 4 National Institute for Health Research Bristol Biomedical Research Centre, University Hospitals Bristol NHS Foundation Trust, Bristol, United Kingdom; 5 Bristol Breast Care Centre, North Bristol NHS Trust, United Kingdom; 6 Division of Informatics, Imaging & Data Sciences, Faculty of Biology, Medicine and Health, University of Manchester, United Kingdom; 7 Division of Cancer Sciences, Faculty of Biology, Medicine and Health, University of Manchester, United Kingdom; 8 Health Education West Midlands, Royal Wolverhampton NHS Trust, United Kingdom; 9 University Hospital Crosshouse, NHS Ayrshire and Arran, United Kingdom; 10 Department of Oncology and Metabolism, University of Sheffield, United Kingdom; 11 James Paget University Hospital, Great Yarmouth, United Kingdom; 12 Rotherham NHS Foundation Trust, Rotherham, United Kingdom; 13 Lincoln County Hospital, United Lincolnshire Hospitals NHS Trust, United Kingdom

**Keywords:** Breast, localisation, surgery, lumpectomy, shared learning, protocol

## Abstract

**Introduction:**

New medical devices must have adequate research, such that outcomes are known, enabling patients to be consented with knowledge of the safety and efficacy of the device to be implanted. Device trials are challenging due to the learning curve and iterative assessment of best practice. This study is designed to pilot a national collaborative approach to medical device introduction by breast surgeons in the UK, using breast localisation devices as an exemplar. The aim is to develop an effective and transferable surgical device platform protocol design, with embedded shared learning.

**Methods and analysis:**

The iBRA-net localisation study is a UK based prospective, multi-centre platform study, comparing the safety and efficacy of novel localisation devices with wire-guided breast lesion localisation for wide local excision, using Magseed® as the pilot intervention group. Centres performing breast lesion localisation for wide local excision or excision biopsy will be eligible to participate if using one of the included devices. Further intervention arms will be added as new devices are CE marked. Outcomes will be collected via an online database. The primary outcome measure will be identification of the index lesion. Participating surgeons will be asked to record shared learning events via online questionnaires and focus group interviews to inform future study arms.

**Ethics and dissemination:**

The study will aim to collect data on 950 procedures for each intervention (Magseed® and wire localisation) from UK breast centres over an 18-month period. Shared learning will be prospectively evaluated via thematic analysis to refine breast localisation technique and to promote early identification of potential pitfalls and problems. Results will be presented at national and international conferences and published in peer reviewed journals.

**Registration:**

This is a UK national audit registered with Manchester University NHS Foundation Trust.

**Highlights:**

- This protocol outlines a novel methodology for a collaborative national platform study to collate safety and efficacy data on new medical devices. Improved registration and audit of new medical devices is a major theme of the Cumberlege report of the Independent Medicines and Medical Devices Safety Review.
- We outline a protocol for a UK based multi-centre prospective audit to investigate the safety and efficacy of new surgical devices for breast lesion localisation. The study will run as a platform study using wire localisation as a control group and Magseed® as the first intervention arm.
- The protocol is designed for additional bolt-on intervention arms for other localisation devices, such as Hologic Localizer™ and Savi Scout®, when they become available to the European market. This will enable comparison of these devices to datasets already collected on wire and Magseed® localisation.
- The study includes a novel shared learning methodology using iterative online database reporting and surgical interviews to centrally distribute information on learning events, critical governance issues and recommended protocols for future use.

## Introduction

Difficulties exist in trials of surgical innovations where the surgeon is learning a new technique and the extent of the risks are unknown [1 2]. The European approval process for a new medical device requires the manufacturer to demonstrate safety of the device [3], but unlike the introduction of a new medicine, there was no requirement for clinical studies to provide ongoing efficacy data once CE marking is given. Device introduction across the United Kingdom (UK) is often driven by the marketing of the product by the manufacturer or the distributor [4]. Trials of the device are usually performed in small numbers in disparate audits or research studies, with variability in outcome measures and quality [5,6].

Platform studies offer a potential solution in the evaluation of new surgical devices, where multiple interventions are evaluated against a common control group within a single protocol [7,8]. The platform design facilitates flexibility for a new experimental arm to be added and for the control arm to be updated during the trial [9], as surgical innovations are developed or iteratively refined. Consequently, multiple interventions can be evaluated in a perpetual manner under a single master protocol [10], which share the same infrastructure with standardised trial procedures. Platform studies are pertinent in the assessment of new surgical devices, where surgical technique or study outcomes may require iterative adjustment.

Breast cancer localisation is an area which has seen rapid development of new surgical devices [11,12,13]. Breast surgery requires the use of multiple medical devices such as meshes, and devices to localise breast lesions for excision. Magseed® [14], Hologic Localiser™ [15] and Savi Scout® [16] are new devices which may offer clinical and logistic benefit over wire-guided breast lesion localisation, but conventional clinical trials evaluating their efficacy are likely to be limited by learning curves and a potential for surgical bias. A multi-arm platform study offers an advantage in allowing new experimental arms to be added, as these new localisation devices or techniques are CE approved.

The IDEAL (idea, development, exploration, assessment, long term study) study framework [17] sets out the stages through which surgical innovations should pass in the assessment of device safety and clinical efficacy. Event reporting of unexpected issues or outcomes associated with a new surgical device or procedure is encouraged [18]. Guidance on how best to capture the evidence on shared learning to guide future surgical practice is limited [19], however, including an assessment of the impact of shared learning during the assessment of new devices on learning curves and clinical outcomes.

The iBRA-net group [20] is a collaborative group of UK breast surgeons, structured as a part of the Association of Breast Surgery, the national body representing breast surgeons. The Association of Breast Surgery and iBRA-net are committed to the evaluation of new devices and techniques in breast surgery. iBRA-net was developed [20] such that a new device could be evaluated by a large group of centres using a common set of outcome measures. The aim is to establish a pathway for new device introduction to collect outcome data on the product, enable shared learning and provide patient information resources to allow true informed consent.

## Aims and objectives

The study protocol is designed to pilot a national collaborative approach to medical device introduction by breast surgeons in the UK, using breast localisation devices as an exemplar. The overall aim is to develop an effective and transferable surgical device platform protocol design, with embedded shared learning to potentially accelerate the learning curve.

The aim of the iBRA-net localisation study is to audit and describe the breast lesion localisation rates across multiple centres in the United Kingdom. In addition, the impact of shared learning on the learning curve and analysis of secondary outcomes will be determined.

The primary outcome is to compare the identification rates of the index lesion in the excised tissue, between women undergoing surgical excision of an impalpable breast lesion with wire guided excision as the control group, and Magseed® localisation, as the primary intervention group. Additional devices will be bolted on as a comparator arm in the platform study when approved for use in the UK and European market.

Key secondary outcomes include:

1. Clinical outcomes related to the localisation device: margin status, accuracy of placement, pathological weight of the specimen, duration of surgery, perioperative complications or adverse events, reoperation rate and cancellations.
2. Shared learning events from qualitative feedback: refinement of clinical outcomes or endpoints, patient selection criteria and surgical approaches during the learning curve.
3. Novel trial design efficacy: qualitative survey assessment of whether shared learning dissemination within the study changed clinical practice or accelerated the learning curve for innovative surgical localisation techniques.
4. Collecting national data to inform current practice of breast localisation techniques in the UK.

## Methods

### Study design

The iBRA-net localisation study is a UK based prospective, multi-centre platform study, with embedded novel shared learning methodology, which will compare the safety and feasibility of breast localisation devices as an exemplar. The study will begin with a National Practice questionnaire, designed with quantitative outcomes to ascertain which devices are used in the UK and qualitative questions to explore what clinicians think about their current localisation technique and what change or improvements they require. The main study will run as a platform cohort study using wire localisation as a control group and Magseed® as the first intervention arm, which already has CE approval. The study protocol is designed to permit additional bolt-on intervention arms as new localisation devices are approved for use in the UK and European healthcare market. This will enable comparison of new devices to data sets already collected on wire and Magseed® lesion localisation.

### Setting

All surgical centres in the United Kingdom performing breast lesion localisation for surgical excision will be invited to participate. Invitations will be advertised through the professional associations, including the Association of Breast Surgery (ABS), British Association of Plastic and Reconstructive and Aesthetic Surgeons (BAPRAS), and Mammary Fold Research Network.

### Participants

#### Inclusion criteria

All female patients over the age of 16 years electing to undergo breast conserving wide local excision or excision biopsy, where localisation is required, will be eligible for inclusion in the initial study.

#### Exclusion criteria

Women will be excluded from the study if there is a contraindication to the localisation device. For instance, for Magseed® this includes:

- They have had an iron oxide injection within the previous six months
- They have a pacemaker or implantable electronic device in situ
- They are unable or do not provide consent for Magseed® localisation
- They are not suitable for general anaesthetic
- They have allergies prohibiting the use of Magseed® localisation

#### Registration

Sites wishing to join the study must complete a registration process. This involves registration of site demographics and identification of a Principal Investigator for each site. The registration process is electronic and will generate a copy of all the necessary study documentation for the Principal Investigator, including the study protocol and patient information documents. Once the study site has local audit approval, access to the electronic database is granted.

#### Recruitment

Potential participants will be identified by the local breast team through breast and oncoplastic clinics, multi-disciplinary team meetings and consultant surgeon or specialist breast research nurse review.

Women will be given a patient information leaflet explaining the Magseed® procedure when this is performed. Participants will be informed of the innovative nature of the device and that outcome data for Magseed® are limited until the results are known. An identical process of consent will be followed as new devices are bolted onto the platform study.

#### Procedural learning and standardisation

Breast centres undertaking a new localisation technique should complete a quality assurance period prior to participation to ensure adequate expertise in radiological placement and surgical removal. Surgeons competent in breast localisation excision will be eligible to participate, but the study requires a minimum standard of surgical competence in performing the procedure with each device to ensure consistent quality in localisation and excision for analysis. It is anticipated that new devices (Magseed®) may require initial training prior to study participation to allow familiarisation with the new device. This may include a trainee with less experience, provided a suitably experienced consultant lead is identified to supervise. The operating surgeon must have completed a minimum of 10 wire-guided wide local excisions within the previous 12 months, and/or a minimum of five Magseed® localisation surgeries, prior to participation in the initial platform study arm.

Scheduling of wire-guided localisation should be performed as per local Trust standard practice. There is a recommended technical procedure for use of the Magseed® to ensure consistent quality in insertion, localisation, and surgical excision (Appendix 1). As patients are recruited and individuals gain expertise in surgical technique, it is planned that technical guidance will be iteratively updated, and the results distributed to participating breast centres via a monthly electronic newsletter.

## Outcomes

### Clinical outcomes

The primary outcome is to evaluate the identification rate of the index lesion in the pathological specimen, comparing the comparator of wire localisation versus the intervention of the new localisation technique.

Secondary clinical objectives which will be assessed include margin status, accuracy of placement, pathological weight of specimen, reoperation rate, cancellations on day of surgery, duration of surgery, complications (e.g. haematoma, infection, wound dehiscence, 30-day readmission, 30-day reoperation, deep vein thrombosis). Patient and tumour data including age, imaging reports, preoperative and postoperative pathology will also be studied. Outcomes for any subsequent arms may be modified to reflect both internal and external scientific discoveries after the initial analysis, once the Magseed® study arm is complete.

## Data collection and management

No patient identifiable data will be reported for the purpose of this cohort study. Patients will be allocated a unique alphanumeric study identification number and all data will be anonymised. Clinical data for each patient will be collected via an online case report form hosted on REDCap (Research Electronic Data Capture). REDCap is a secure, web-based database designed for use in collaborative clinical research [21] and hosted by the University of Oxford. Access is limited to study executives via a password protected account and all web-based information is encrypted.

Online case report forms used to capture data on REDCap will be divided into eight domains (identifiers, preoperative radiology, preoperative oncology, localisation data, pathology, perioperative complications, 30-day complication data, shared learning events). Shared learning inputs will comprise of a ‘yes/no’ prompt followed by a free text box to allow qualitative elaboration.

## Data analysis

### Sample size

Power calculations estimate 950 patients per group sufficient to establish equivalence between Magseed® and wire-guided localisation for the initial study arm, based on an upper limit of observed one-sided 95% confidence interval for a difference between failure rates expected to be less than 0.9%, with 80% power.

### Statistical analysis

All data analysis will be conducted centrally using standard statistical software (e.g. SPSS) and will be led by the University of Manchester. Simple descriptive summary statistics will be calculated to describe the main parameters and variations in practices of breast localisation technique. Categorical data will be summarised by counts and percentages, and continuous data by the mean or median and their associated measures of dispersion, dependent on the distribution of the data [22]. Regression analysis will be used to control for predictive variables. Differences between groups using unpaired t-tests, Mann-Whitney U tests and Chi squared tests, as appropriate. Any qualitative data, which comprises free text responses to open ended items from the online case report forms, will be presented according to overall themes using a thematic or content analysis as appropriate [23].

### Interim analysis

Interim analyses will be undertaken when a total of 400 patients from a minimum of 10 centres have been recruited to the study. Centres with an overall index lesion identification rate audited as an outlier (>3SD from control) will be contacted to check the validity of the results, explore potential reasoning behind this (e.g. learning curve and training requirement) and escalated as per local hospital trust protocol if persistently anomalous (>3 SD).

### Patient and public involvement

Oversight of the study will be led by the iBRA-net audit Steering Committee which has representation from surgeons, trainees, patient representatives, and academics with experience of study management and statistics. This group meets twice a year, with additional executive meetings arranged as required via e-mail or teleconferencing. Regular monthly auditing to review study progress, protocol compliance and dissemination of technical recommendations will be overseen by the study executive committee.

The overall results from the study will be discussed with the iBRA-net Study Group collaborative to inform the planning and design of the next phase of the platform trial, provided the evidence shows that Magseed® meets the required safety standards [24] and as new surgical devices emerge.

## Shared learning

Central incident reporting is encouraged for unanticipated learning events or issues with new surgical devices [25]. Shared learning will be achieved in two phases using a novel mixed-method approach to explore the feasibility of each technique by asking participating surgeons to complete online +/- face-to-face shared learning.

### Online shared learning

Prospective shared learning documentation will be collected for each patient using the online case report form on REDCap for the duration of the study. Surgeons will be prompted to identify problems related to device insertion (e.g. insertion of the localisation device >2cm from the index lesion), perioperative issues before the induction of anaesthesia (e.g. percutaneous failure to localise lesion) or intraoperative events (e.g. failure to remove index lesion). A free-text box will be used in which clinicians may expand on the nature of the shared learning event and to document any technical tips applied to overcome them.

The iBRA-net localisation steering committee will regularly review shared learning events and feedback to study participants to allow iterative improvement of surgical technique [26].

### Focus group interviews

A purposive sample of participating breast surgeons and radiologists will be invited to participate in a focus group interview to further discuss any shared learning points raised in the online database and to refine any technical modifications outlined in participating UK breast centres. Interviews will be conducted using a semi-structured topic guide based on the online shared learning outcomes, including the complications identified, rationale and clinical outcome. Data will be analysed via thematic analysis and participating surgeons will be invited to review the final outcomes to ensure a valid reflection of learning events is reported.

### Evaluation of shared learning

Shared learning from the online database will be summarised thematically. Major technical modifications or learning events identified will disseminated in a timely fashion via a monthly electronic newsletter update to participating surgeons during the study, to enable iterative sharing of technical tips, potential pitfalls and to accelerate the learning curve.

Overall evaluation of the online database and focus group shared learning will be undertaken at the end of the first localisation device (Magseed®) study arm to inform the subsequent arms of the platform study. A thematic analysis will be conducted independently by >3 co-authors of both the written and interview qualitative data to identify common learning events, triangulate key findings identified from each method and to ascertain how each shared learning approach may have impacted upon surgical practice.

Additional qualitative feedback may be sought from all participants via an electronic questionnaire to determine the number of surgeons who used shared learning to inform their clinical practice, and to aid an evaluation of the value of each of the shared learning methodologies applied, to iteratively inform the next phase of the study.

## Dissemination of results

The platform study results will be propagated through national and international presentation and publication in peer reviewed journals. All presentations and publications will be made on behalf of the UK Surgical Trainee Research Collaborative and iBRA-net Study Group collaborative. Study centres will be presented with a summary of the study data in the form of a webinar and access to the published manuscript.

The results of this study can be used for future consent of patients receiving localisation devices, ensuring their consent is informed of the likely outcomes associated with the device in multi-centre practice.

Results from the shared learning analysis will be used to inform the subsequent arms of the platform study and may be transferable to other surgical trials of new devices or techniques.

## Appendix 1

### i) Magseed® user summary

- Magseed® is licenced to be placed up to 30 days in advance of operation date.
- Magseed® should not be placed until all investigations have been completed, e.g. post neoadjuvant chemotherapy magnetic resonance imaging.
- Patients should receive an information leaflet about Magseed® prior to insertion.
- Prospective outcomes of Magseed® localisation should be audited.
- Absolute exclusion criteria for using Magseed®:
  - Patients with a Pacemaker or implanted device in the chest wall
  - Patients requiring an MRI scan between Magseed® placement and surgery
  - Patients who have received Sienna (iron oxide) injection in the previous six months
- Caution criteria for using Magseed®:
  - Metal coronary stents.
  - Failure to locate Magseed® in anaesthetic room prior to anaesthetic induction
  - Failure to locate and differentiate between Magseed® clips if multiple seeds are used for bracketing lesions that are close together.
- Ensure Sentimag® device is switched on in theatre at least 20 minutes prior to first use to allow sufficient time to warm up and identify faults.
- Wire localisation can continue to be available when clinicians feel that this would be preferable for an individual patient.

### ii) Magseed® user technical guidelines

1. Connect the probe with the base unit ensuring that the arrows on the probe connectors are at the top of the connectors
2. Switch on the Sentimag at least 15–20 minutes prior to use. The dial needs to be set at position 2 throughout the procedure.
3. Cover the probe with a sterile single-use sheath
4. Balance the Sentimag using the balance button or the footswitch Probe.
5. The operator should always hold the probe behind the black ring. Make sure all metal including rings, retractors, lights, name badges are out of the range of the probe.
6. To perform a balance of the base unit, the operator should either press the button marked on the base unit or press the footswitch. After five seconds the scales symbol will stop and the Sentimag should display a value close to zero. Scales will require balancing when the stationary balance symbol is displayed (e.g. after start-up), when the sensitivity setting of the Sentimag is changed, before starting use after a minimum of 15 minutes warm-up, before taking any measurements on the patient.
7. For transcutaneous measurement, sweep the probe and apply some pressure around the breast until the Magseed® is located (to get a signal from Magseed® the probe must be within 3cm). Pivot the probe around the hotspot to maximize the signal and pinpoint the lesion.
8. Confirm the tracing of the Magseed®. Palpation of the skin should result in a rise and fall in the signal = a characteristic change in Sentimag value and audio frequency. The signal will increase when the probe is pointing directly at a Magseed® lesion and decrease when angled away.
9. Pin-point technique. Remove probe from incision, balance in air and recheck suspect lesion.
10. Balance in-vivo: From within the incision, withdraw 2–3 cm from the suspect lesion and re-balance. A clear positive signal should be seen when you examine the Magseed® lesion again.

For more information please visit: *https://www.endomag.com/Magseed/overview/*.
